# Decoding immune cell dynamics in ischemic stroke: insights from single-cell RNA sequencing analysis

**DOI:** 10.3389/fnagi.2025.1549518

**Published:** 2025-04-15

**Authors:** Yating Lan, Chun Zou, Feiyu Nong, Qi Huang, Jingyi Zeng, Wenyi Song, Guining Liang, Qingyan Wei, Mika Pan, Donghua Zou, Yaobin Long

**Affiliations:** ^1^Department of Neurology, The Second Affiliated Hospital of Guangxi Medical University, Nanning, Guangxi, China; ^2^Department of Rehabilitation, The Second Affiliated Hospital of Guangxi Medical University, Nanning, Guangxi, China

**Keywords:** ischemic stroke, immune cells, single cell analysis, phagocytosis, enrichment analysis, differential gene expression, cell communication

## Abstract

**Background:**

Ischemic stroke (IS) is a leading cause of adult disability worldwide. The inflammatory processes involved are complex, making it challenging to fully understand the pathological mechanisms of IS. Phagocytosis plays an important role in eliminating neurotoxic or damaged neurons resulting from inflammatory responses. This study employed bioinformatics methods to analyze single-cell RNA sequencing (scRNA-seq) data to investigate the cell types and molecular biological processes involved in IS.

**Methods:**

scRNA-seq data for IS were obtained from the Gene Expression Omnibus (GEO). Following sample screening and reprocessing, 5,582 single cells were identified from healthy controls and patients with IS. Uniform manifold approximation and projection (UMAP) was utilized to further explore the cellular composition in IS. Functional enrichment analysis of differentially expressed genes was conducted to identify transcriptional regulators, whereas cell developmental trajectories were predicted to uncover potential cell fate decisions. iTALK was employed to identify potential ligand-receptor axes within the cell-type immune microenvironment of IS.

**Results:**

Based on scRNA-seq data analysis, we identified four cell types and their associated subclusters, along with genes exhibiting significant differential expression within these subclusters. Phagocytosis was significantly enriched in cell types linked to IS, while the differentiation trajectories of subpopulations in IS was different. Additionally, multiple receptor-ligand axes were identified, indicating diverse interactions within the immune microenvironment of IS.

**Conclusion:**

This study demonstrated that phagocytosis in IS cell types critically influences disease progression. It also predicted the trajectories of infarct cells. These findings provide valuable insights into the molecular and cellular mechanisms underlying IS and highlight potential pathways for therapeutic intervention.

## 1 Introduction

Stroke is the second leading cause of disability and death worldwide, imposing a significant burden on individuals and society. Total direct medical expenses related to stroke are projected to more than double between 2015 and 2035, increasing from $36.7 billion to $94.3 billion (Saini et al., 2021). In China, stroke is a leading cause of death and loss of disability-adjusted life years, with ischemic stroke (IS) comprising approximately 65.3% of all stroke cases (GBD 2021 Nervous System Disorders Collaborators, 2024; Ma et al., 2021). The primary clinical manifestations of IS include sudden weakness or numbness on the contralateral limb of lesions, difficulty in speaking or understanding speech, impaired consciousness, balance or coordination challenges, and vision loss (Walter, 2022). Cerebral infarction is characterized by high rates of morbidity, disability, mortality, and recurrence. The annual incidence of cerebral infarction is approximately 0.2% in the general population. Generally, around 15 million people experience cerebral infarction annually, with 5 million dying from the condition and another 5 million permanently losing their ability to work (Donkor, 2018). Early identification of acute IS, prompt interventions to restore blood flow, and timely treatment in stroke centers can significantly reduce morbidity and mortality (Herpich and Rincon, 2020). Effective treatment of cerebral infarction requires strict adherence to time-sensitive protocols (Mendelson and Prabhakaran, 2021). Additionally, IS triggers a complex inflammatory cascade that contributes to localized brain damage (Petrovic-Djergovic et al., 2016). However, the exact molecular mechanisms underlying this process remain unclear.

Phagocytosis initiates a cascade of inflammatory responses in the brain during IS that protects neurons and improves disease prognosis. Conversely, excessive clearance can exacerbate neuronal damage and cerebral infarction. Inflammation is mainly triggered by resident immune cells, like microglia, astrocytes and others, besides, classical immune cells such as neutrophils, macrophages, and T lymphocytes also participate in this process (Wang H. et al., 2023; Candelario-Jalil et al., 2022). Recruitment of peripheral immune cells occurs through the release of chemokines at the site of injury, which activate endothelial cells and subsequently disrupt the blood–brain barrier (Blank-Stein and Mass, 2023). Studies have found that after cerebral infarction, microglia in the brain rapidly detect danger signals and participate in the immune response. Subsequently, macrophage-like cells, neutrophils, pro-inflammatory cytokines, tumor necrosis factor, interleukins, and other immune mediators are activated. These elements contribute to the regulation of immune signaling and the recognition of dead cells, pathogens, and autoantigens (Lambertsen et al., 2019). CD4 + T lymphocytes play a critical role in mediating tissue damage after IS. Animal experiments have shown that CD4 + T cell-mediated responses promote B cell infiltration into the central nervous system following cerebral infarction, likely involving additional interactions with microglia and infiltrating peripheral myeloid cells (Weitbrecht et al., 2021). Macrophage phagocytosis involves recognizing, binding, engulfing, and digesting apoptotic cells. This process prevents secondary necrosis caused by tissue damage and inflammation while promoting pro-resolving signaling in macrophages, which is essential for tissue decomposition and repair (Schilperoort et al., 2023). Naïve T cells and T cells are recruited during the formation of atherosclerotic plaques to regulate macrophage polarization by secreting pro-inflammatory and anti-inflammatory factors. These macrophages destabilize atherosclerotic plaques by secreting pro-inflammatory factors (Carrasco et al., 2022). Natural killer (NK) cells are detrimental to chronic inflammation and autoimmune diseases. Their accumulation and increased cytotoxic potential induce apoptosis or necrosis of endothelial cells in the vascular wall, which promotes unstable atherosclerosis. However, NK cells also exert a protective effect on cells damaged by cerebral infarction by mitigating their cytotoxic effects (Crinier et al., 2020; Kyaw et al., 2017). Thus, further exploration of the biological functions of various cell types and the regulatory mechanisms of associated transcriptional factors is important for a deeper understanding of the pathological progression of IS. This may provide new insights into the diagnosis and treatment of cerebral infarction, emphasizing the need for detailed investigations into the relationship between cell subtypes and their biological roles in IS.

With the rapid advancement of science and technology, next-generation sequencing (NGS) technology has evolved significantly from genomics, transcriptomics, and epigenomics to single-cell characterization. This progression has attracted substantial attention for uncovering more nuanced discoveries (Hwang et al., 2018). Single-cell RNA sequencing (scRNA-seq) facilitates the analysis of cell heterogeneity and reveals regulatory relationships between genes. scRNA-seq of immune cells is widely employed to comprehensively analyze the immune system (Papalexi and Satija, 2018).

This study aimed to investigate the cell types and molecular biological processes involved in IS. We used scRNA-seq to investigate the cell types involved in IS, examine the IS ecosystem, and identify related cell subclusters. Additionally, we explored the developmental trajectories, intercellular communication, and signaling pathways within these IS cell subpopulations. Finally, we identified specific biomarkers that maintain homeostasis in IS and examined the interactions between different cell types. Our findings offer deeper insights into the cellular functions and key signaling pathways associated with IS, potentially providing innovative strategies for its diagnosis and treatment.

## 2 Materials and methods

### 2.1 Data acquisition and processing

We obtained the scRNA-seq dataset GSE224273 (Fernandez et al., 2019) from IS and healthy individuals via the Gene Expression Omnibus (GEO).^1^ This dataset was based on the GPL20301 platform and included nine samples: four carotid atherosclerotic tissue specimens collected during carotid endarterectomy from two IS patients (average age: 70 years) and five specimens from four non-IS patients (average age: 69.75 years). Samples GSM7018585, GSM7018586, and GSM7018587, which did not meet the study’s criteria, were excluded. The final dataset comprised six samples: GSM7018579, GSM7018580, GSM7018581, GSM7018582, GSM7018583, and GSM7018584.

### 2.2 Single-cell RNA sequencing cell clustering

Cells derived from the original dataset underwent standardized data processing and rigorous quality control, including the removal of double cell counts, dead cells, and mitochondrial gene content. The default filtering criteria included cells with a feature count of less than 1% and mitochondrial content greater than 10% of total needs. The filtered high-quality cells were normalized using the sctransform function from the Seurat package in R, sctransform is based on regularized negative binomial regression. First, we constructed generalized linear model (GLM), for each gene with a GLM, the independent variable is the sequencing depth of each cell (total UMI count), and the dependent variable is the UMI count of each gene in each cell. Next, regularization parameter estimation is applied to regularize the parameters in GLM to reduce the effects of overfitting and noise. Then we calculated the residuals using regularized parameters and the sequencing depth of the cells, and recalculate the expected expression levels of each gene in each cell. And subtracted the actual expression amount from the expected expression amount to obtain the residual term. These residual terms reflect true biological heterogeneity in gene expression, not technical variation. Finally, we obtained the standardized expression level of each gene via dividing the residual term by the standard deviation of the negative binomial distribution through variance stabilization transformation, which is the Pearson residual (Hafemeister and Satija, 2019). Furthermore, we constructed a single-cell atlas and cell clustering was performed using the Seurat package in R with default parameters (Butler et al., 2018). The IntegrateData function was employed to integrate all single-cell datasets and the FindClusters function was used to identify clusters. The clustering results were uniformly reduced and visualized using the uniform manifold approximation and projection (UMAP) for the dimensionality reduction algorithm (Becht et al., 2018). The FindAllMarkers function from Seurat was used to identify highly expressed marker genes in each cell cluster. Cell-type annotations were performed using a single-cell atlas based on existing cell-type marker genes (Fernandez et al., 2019).

### 2.3 Differential gene expression analysis

To explore variations in gene expression across different cell types, differential gene expression analysis was performed based on the FindMarkers function in the Seurat package (Butler et al., 2018). This function was used to identify differentially expressed genes between the control and IS groups. Genes with a *p*-value < 0.05 were considered statistically significant.

### 2.4 Functional enrichment analysis

Gene Ontology (GO) and Kyoto Encyclopedia of Genes and Genomes (KEGG) enrichment analysis were applied to determine the molecular pathways and potential functions in each cell subset. We used the clusterProfiler package in R to perform an enrichment analysis of GO biological processes (BP), cellular components (CC), molecular functions (MF), and KEGG signaling pathways on multiple grouped gene lists (Yu et al., 2012). Pathways with a *p*-value < 0.05 were considered significantly associated with marker genes. The Benjamin-Hochberg method was applied to adjust *p*-values and control the false discovery rate (FDR) within an acceptable range (Korthauer et al., 2019). Additionally, global gene expression profiles were analyzed using gene set enrichment analysis (GSEA) to explore potential biological characteristics (Subramanian et al., 2005), with a significance threshold of *p* < 0.05.

### 2.5 Single-cell trajectory analysis

RNA velocity analysis was used to infer the differentiation trajectory of cells during IS by linking measurements with potential mRNA splicing dynamics (Manno et al., 2018). We used the velocyto software which based on python, firstly, the single-cell data was performed by cellranger, then we used the steady-state/deterministic models to infer gene dynamics from the abundance ratio between spliced and unspliced mRNA to derive RNA velocities that can be used to infer the evolutionary trajectory of cells in IS pathogenesis (Bergen et al., 2020; Svensson and Pachter, 2018).

### 2.6 Construction of gene regulatory network

Single-cell regulatory network inference and clustering (SCENIC) was used to construct gene regulatory networks and identify cell states based on single-cell expression profiles, providing important insights into the mechanisms driving cellular heterogeneity. To analyze the intrinsic transcriptional regulatory drivers of IS, we used the Python module tool pySCENIC to reconstruct gene regulatory networks centered on transcription factors (TFs) (Aibar et al., 2017; Van de Sande et al., 2020).

### 2.7 Cell communication analysis

Signal transduction emphasizes the mode and results of signal reception, transmission, and the conversion of signals between cells, with ligand-receptor binding being a primary mechanism. In this study, the iTALK package in R was used to identify high-confidence ligand-receptor interactions between cells (Wang et al., 2019). It preferentially identifies highly expressed or differentially expressed genes in cell clusters and matches them with entries in a ligand-receptor database to identify significant intercellular communication events. The interaction ring network diagrams and differential interaction ring diagrams table were generated to visualize these interactions.

### 2.8 Data analysis and statistics

All bioinformatics analyses in this study were conducted using the Bioinforcloud platform.^2^ Differential gene expression levels were evaluated with a non-paired *t*-test. Statistical significance was indicated with a *p*-value < 0.05.

## 3 Results

### 3.1 Global single-cell landscape of IS and healthy controls

**Figure 1:**
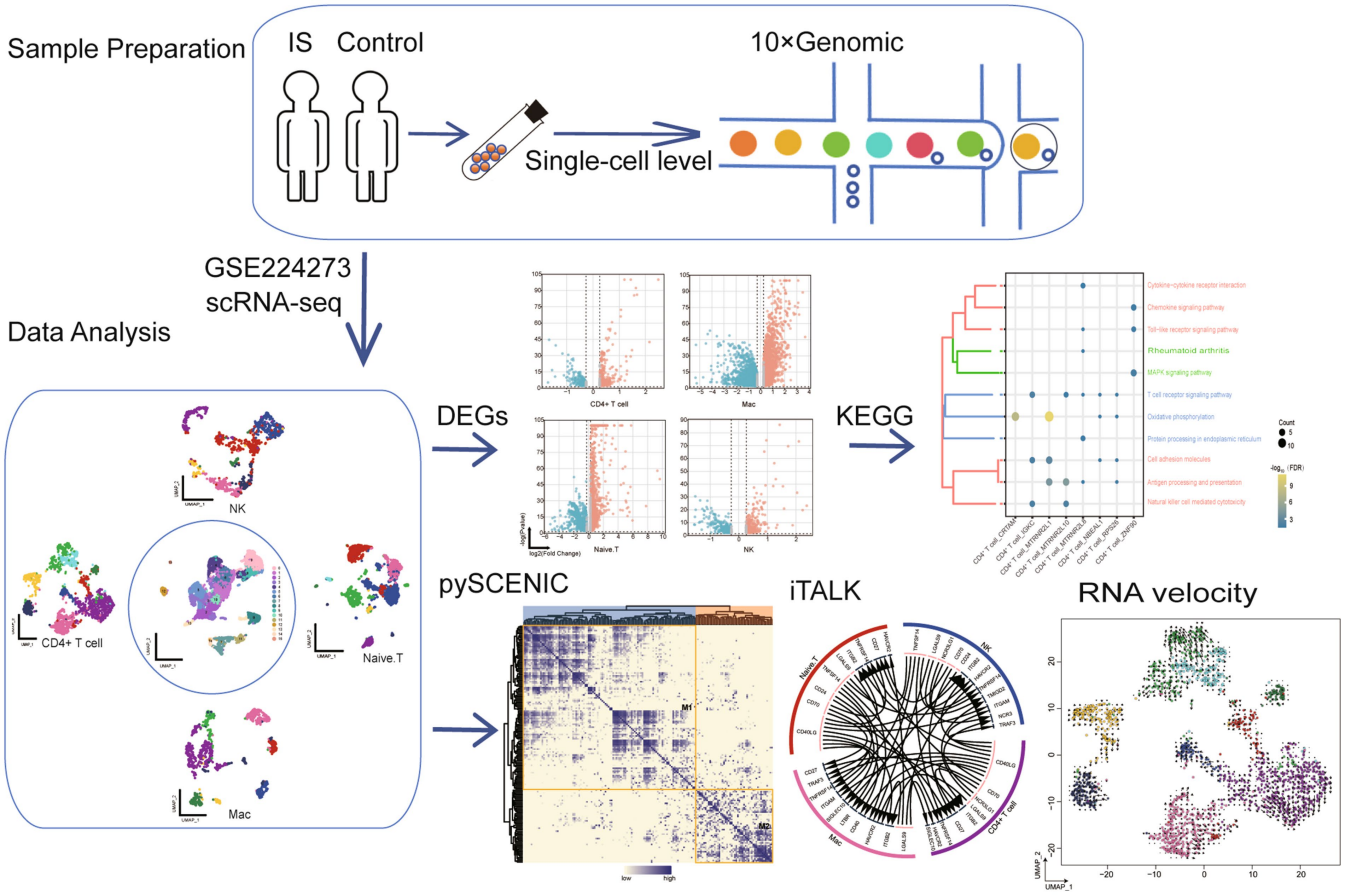
Flowchart of this study. IS, ischemic stroke; scRNA-seq, single-cell RNA sequencing; NK, natural killer cells; Mac, macrophages; Naive T, naive T cells; DEG, different expression gene; KEGG, Kyoto Encyclopedia of Genes and Genomes; UMAP, Uniform manifold approximation and projection.

**Figure 2:**
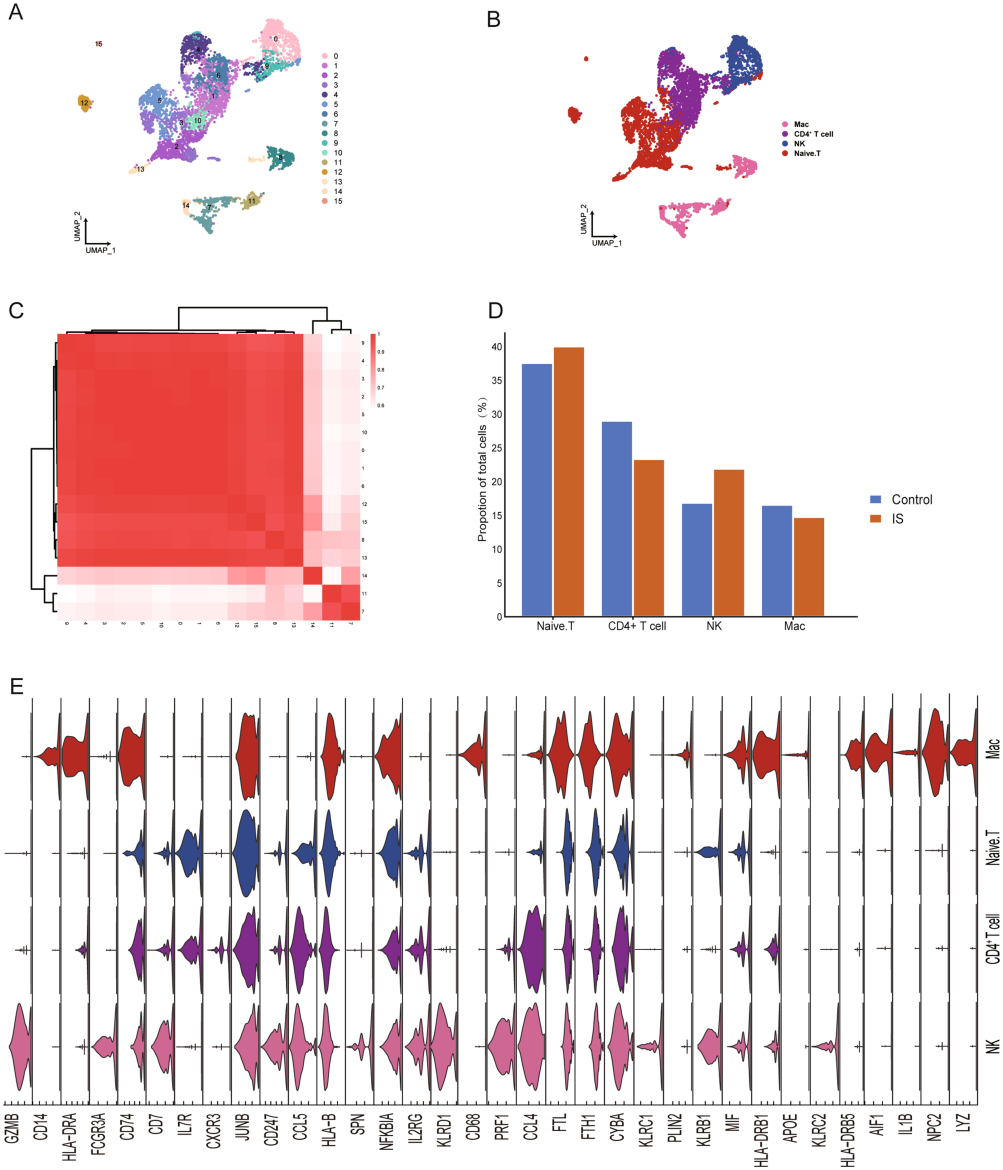
Global single-cell atlas of IS. **(A)** The mononuclear atlas identified 15 cell clusters. **(B)** IS mapping of single-cell types: CD4 + T cells, Mac, Naive T, NK. **(C)** Correlation analysis between sample groups. **(D)** Proportions of four cell types in patients with IS and the control group. **(E)** Violin plot displaying characteristic genes in different cell types. UMAP, Uniform Manifold Approximation, and Projection.

To explore the cellular landscape of IS, we analyzed the scRNA-seq dataset retrieved from the GEO database (Fernandez et al., 2019). The workflow of this study is illustrated in Figure 1, with clinical information for all samples provided in Supplementary Table S1. Following standardized data processing and quality control, 5,582 high-quality single-cell transcription profiles were identified and categorized into 15 different clusters (Figure 2A). To further analyze these cell subtypes, we annotated each cell cluster based on the expression of known cell-type markers and used the FindAllMarkers function in R to pinpoint specific marker genes of the annotated cell types (Supplementary Table S2). These 15 clusters were classified into four distinct cell types, encompassing both IS and control groups (Figures 2B,E; Supplementary Table S3). In addition, correlational analysis was conducted among the cell clusters. Using the expression patterns of the different clusters, we calculated the correlations between them (Figure 2C). We then analyzed the abundance of cell types and found an increased abundance of naïve T cells and NK cells in IS, whereas the abundance of CD4 + T cells and macrophages (Mac) decreased in IS (Figure 2D; Supplementary Figure 1).

### 3.2 Biological functions and signaling pathways characteristics of CD4 + T cells

**Figure 3:**
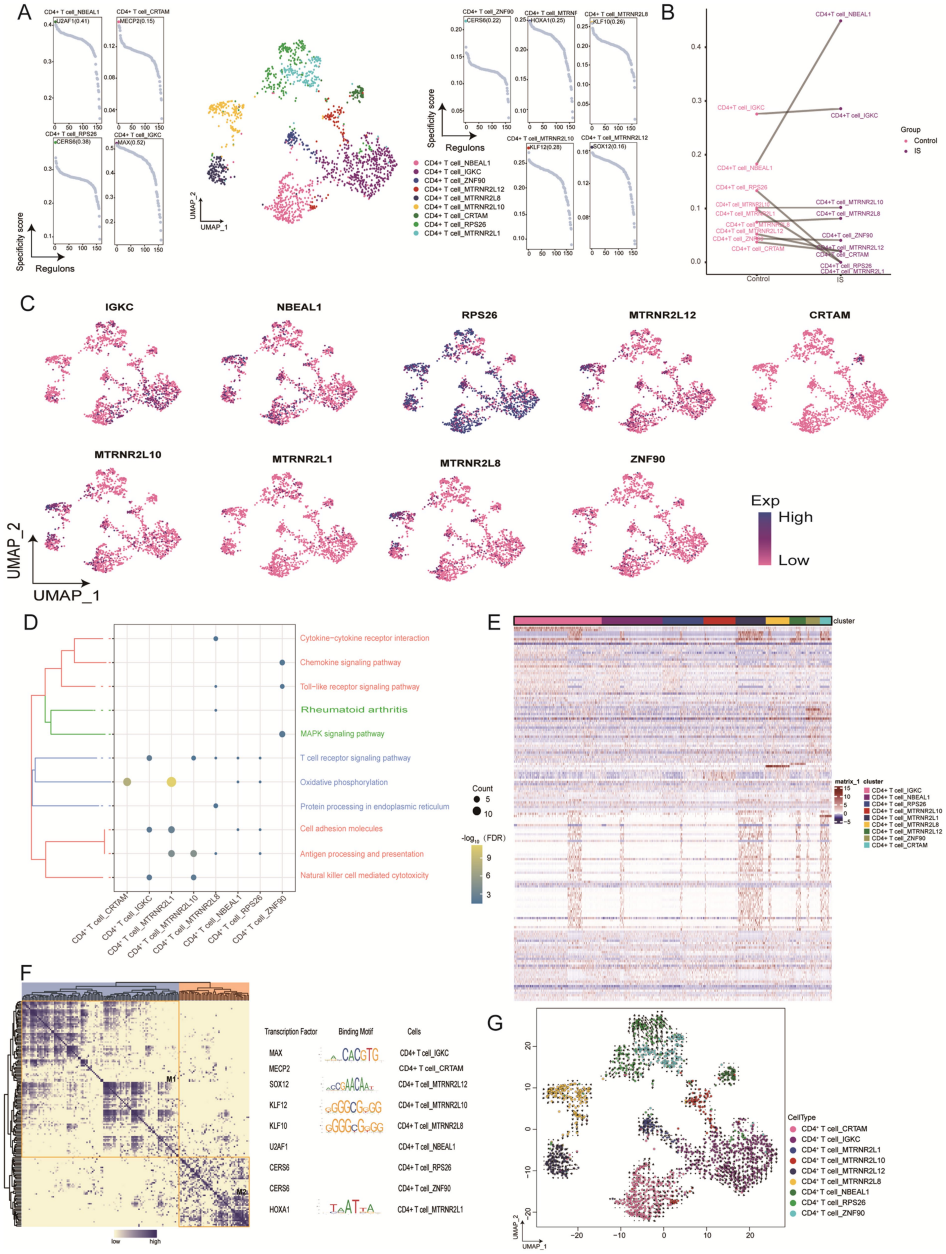
Identification of CD4 + T cell clusters in IS. **(A)** Single-cell plot of CD4 + T cell clusters and scatter plot of subclusters **(B)** Mapping of marker genes in CD4 + T cell subsets. **(C)** Comparison of cell abundance between patients with IS and controls. **(D)** Biological pathway enrichment of CD4 + T cell subsets, with darker colors indicating more prominent enrichment. Count represents the number of genes enriched in a certain pathway, and the larger number, the more genes were enriched in that pathway. -Log10 (FDR) represents the significance of enrichment differences, with darker colors indicated more significant enrichment differences. **(E)** Heatmap of differentially expressed genes in CD4 + T cell subsets. **(F)** Heatmap of motif-based transcription factor (TF) gene regulatory networks for CD4 + T cell subsets. **(G)** Mapping of CD4 + T cells and differentiation trajectories of monocytes, arrows represent the direction of differentiation, and the length of arrows represents the speed of differentiation. UMAP, Uniform Manifold Approximation and Projection.

CD4 + T cells were divided into nine sub-clusters (Figure 3A), all of which were observed during IS. We found that neurobeachin like 1 (NBEAL1), IGKC, and MTRNR2L8 were significantly enriched in IS, whereas MTRNR2L12, CRTAM, RPS26, and MTRNR2L1 were downregulated (Figure 3B). Moreover, the expression levels of these nine genes revealed high RPS26 expression (Figure 3C). In the enrichment analysis, oxidative phosphorylation was upregulated in the CD4 + T cell_CRTAM and CD4 + T cell_MTRNR2L1 subclusters. In contrast, pathways such as cytokine-cytokine receptor interaction, chemokine signaling, toll-like receptor signaling, MAPK signaling, T cell receptor signaling, protein processing in the endoplasmic reticulum, cell adhesion molecules, antigen processing and presentation, and NK cell-mediated cytotoxicity were downregulated. Notably, oxidative phosphorylation was significantly upregulated in the CD4 + T cell_MTRNR2L1 subcluster, whereas chemokine signaling, MAPK signaling, and NK cell-mediated cytotoxicity were significantly downregulated (Figure 3D; Supplementary Table S4). The expression heatmap of markers of specific cell subpopulations depicted gene expression patterns across subclusters (Figure 3E). Within the gene regulatory network for specific markers in these subpopulations, MAX, SOX12, KLF12, KLF10, and HOXA1 emerged as transcriptional regulators (Figure 3F). In addition, RNA velocity analysis of the CD4 + T cells showed that there were two differentiation fate, one was that CD4 + T cell_MTRNR2L1 could differentiate into CD4 + T cell_MTRNR2L8, another was differentiated into CD4 + T cell_IGKC, and the latter differentiated faster than the former (Figure 3G).

### 3.3 Differential gene expression and biological functional landscape of mac

**Figure 4:**
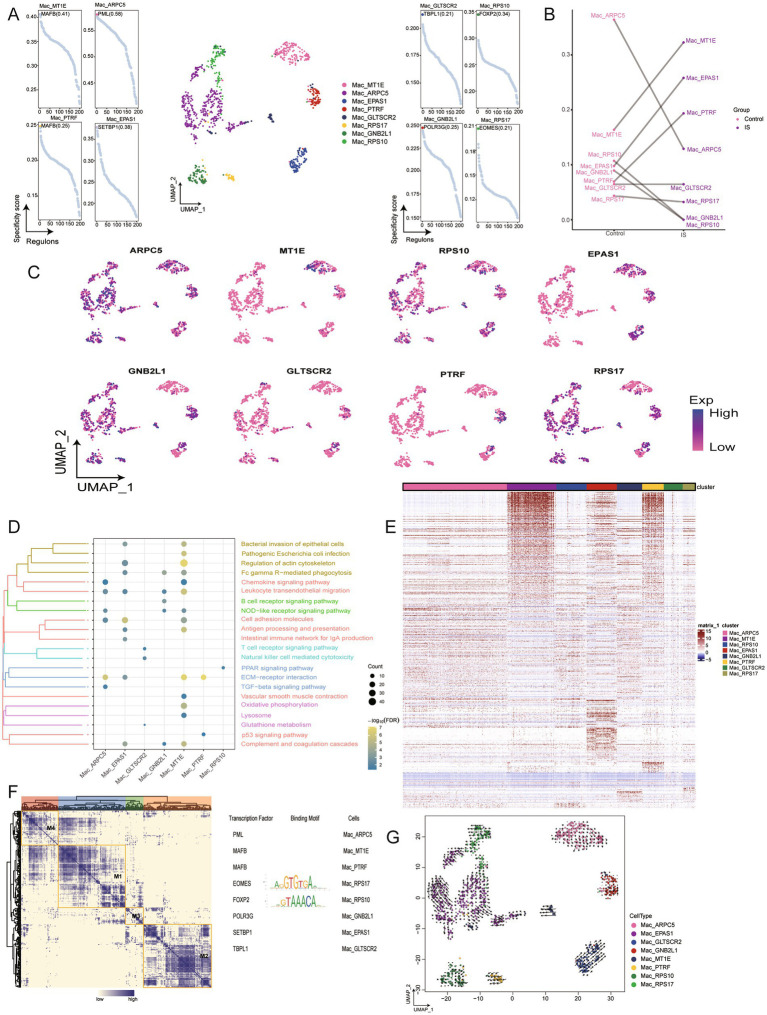
Identification of Mac clusters in IS. **(A)** Single-cell plot of Mac clusters and scatter plot of subclusters. **(B)** Mapping of marker genes in Mac subclusters. **(C)** Comparison of cell abundance between patients with IS and controls. **(D)** Biological pathway enrichment of Mac subpopulations, with darker colors indicating more prominent enrichment. Count represents the number of genes enriched in a certain pathway, and the larger number, the more genes were enriched in that pathway. −Log10 (FDR) represents the significance of enrichment differences, with darker colors indicated more significant enrichment differences. **(E)** Heatmap of Mac subclusters. **(F)** Heatmap of motif-based transcription factor (TF) gene regulatory networks of Mac subpopulations. **(G)** Mapping of Mac and differentiation trajectories for monocytes, arrows represent the direction of differentiation, and the length of arrows represents the speed of differentiation. UMAP, Uniform Manifold Approximation and Projection.

Mac were classified into eight sub-clusters (Figure 4A), all of which were observed in IS samples. We found that MT1E, EPAS1, and PTRF were highly enriched, whereas actin related protein 2/3 complex subunit 5 (ARPC5), GNB2L1, RPS10, and RPS17 showed reduced abundance (Figure 4B). Among the eight genes, ARPC5, GNB2L1, and RPS17 exhibited the highest expression levels (Figure 4C). Enrichment analysis revealed that the chemokine signaling pathway was significantly downregulated in the Mac_ARPC5 and Mac_MT1E subclusters. Additionally, Mac_MT1E, which is involved in multiple signaling pathways, showed a strong positive correlation with the regulation of the actin cytoskeleton. Subclusters Mac_ARPC5, Mac_MT1E, and Mac_PTRF were positively associated with the ECM-receptor interaction pathway (Figure 4D; Supplemental Table S5). The expression heatmap of the markers of specific cell subpopulations showed the gene expression patterns (Figure 4E). The gene regulatory network of Mac subclusters identified EOMES and FOXP2 as transcriptional regulators (Figure 4F). Furthermore, the differentiation potential of Mac subclusters in IS was identified by cell trajectory analysis. It was found that Mac_ARPC5 developed into Mac_RPS10, and some cell subclusters had no obvious differentiation fate (Figure 4G).

### 3.4 Biological functional landscape and differentiation trajectory of T cells

**Figure 5:**
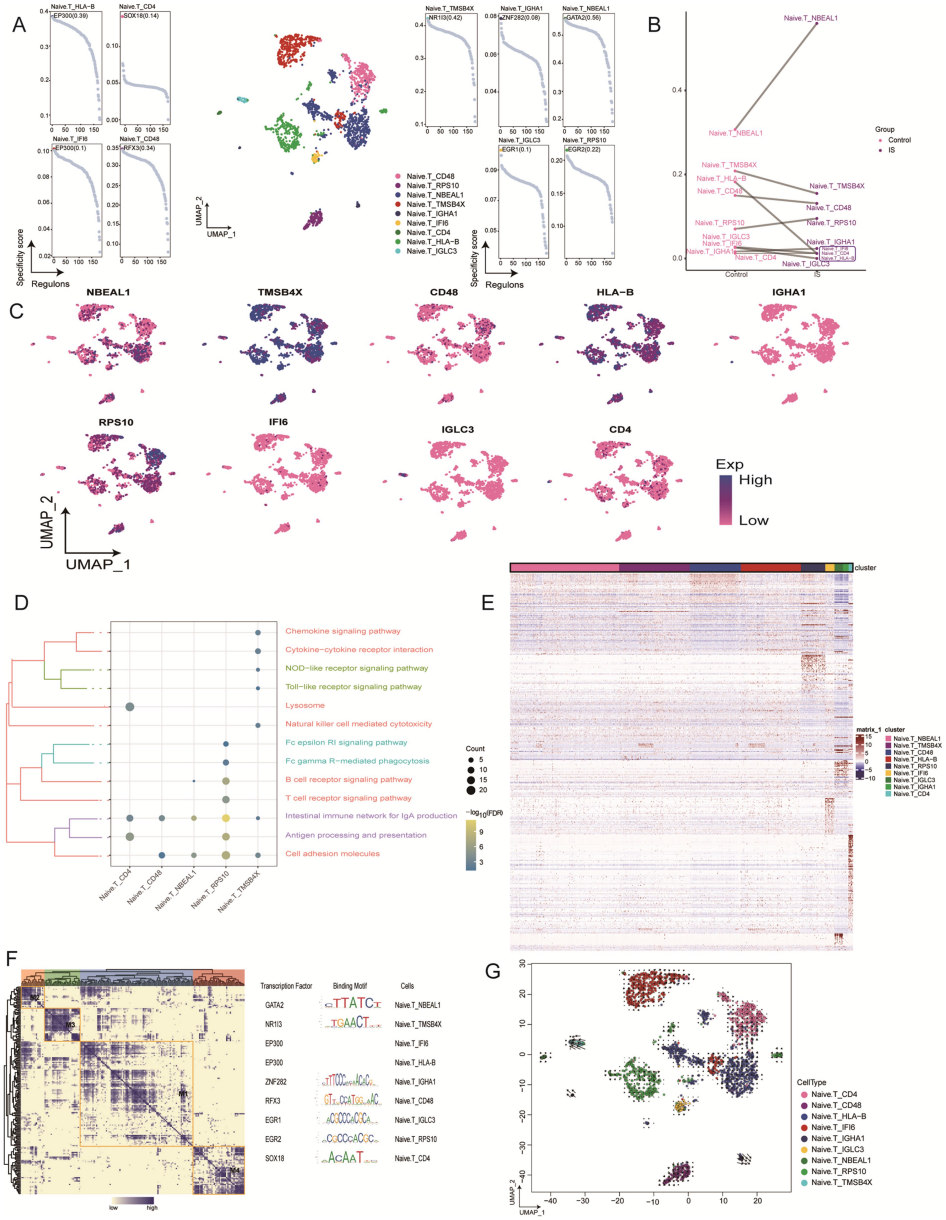
Identification of naive T cell clusters in IS. **(A)** Single-cell plot of naive T cell clusters and scatter plot of subclusters. **(B)** Mapping of marker genes in naive T cell subclusters. **(C)** Comparison of cell abundance between patients with IS controls. **(D)** Biological pathway enrichment of naive T cell subclusters, with darker colors indicating more significant enrichment. Count represents the number of genes enriched in a certain pathway, and the larger number, the more genes were enriched in that pathway. -Log10 (FDR) represents the significance of enrichment differences, with darker colors indicated more significant enrichment differences. **(E)** Heatmap of differentially expressed genes in naive T cell subclusters. **(F)** Heatmap motif-based transcription factor (TF) gene regulatory networks for naïve T cell subclusters. **(G)** Mapping of naive T cells and differentiation trajectories for monocytes, arrows represent the direction of differentiation, and the length of arrows represents the speed of differentiation. UMAP, Uniform Manifold Approximation and Projection.

Naïve T cells were categorized into nine subclusters (Figure 5A), all of which were identified in patients with IS. We found that NBEAL1 and RPS10 were significantly enriched in IS, whereas HLA-B, TMSB4X, CD48, IGLC3, and IFI6 were downregulated (Figure 5B). Furthermore, we determined the expression levels of these nine genes and found that TMSB4X and HLA-B were expressed at notably higher levels (Figure 5C). Enrichment analysis revealed that naïve T_RPS10 was involved in multiple signaling pathways, showing a significant positive correlation with the intestinal immune network for IgA production. In contrast, the B cell receptor signaling pathway, Fc gamma R-mediated phagocytosis, Fc epsilon RI signaling pathway, lysosome activity, Toll-like receptor signaling, NOD receptor signaling, cytokine-cytokine receptor interaction, and chemokine signaling pathway all exhibited negative regulatory effects in the naïve T_CD4, naïve T_CD48, and naïve T_TMSB4X subclusters (Figure 5D; Supplementary Table S6). The expression heatmap of specific cell subpopulation markers demonstrated the expression patterns of each gene (Figure 5E). In the gene regulatory network, GATA2, NR1I3, ZNF282, RFX3, EGR1, EGR2, and SOX18 emerged as key transcriptional regulators of the naïve T subcluster (Figure 5F). And exploring the changes in cell state of Naive.T subclusters in IS through RNA velocity analysis, it was suggested that Naive.T_NBEAL1 transformed into Naive.T_CD48, although the entire differentiation process was slow (Figure 5G).

### 3.5 Differential gene expression and biological functional landscape of NK cells

**Figure 6:**
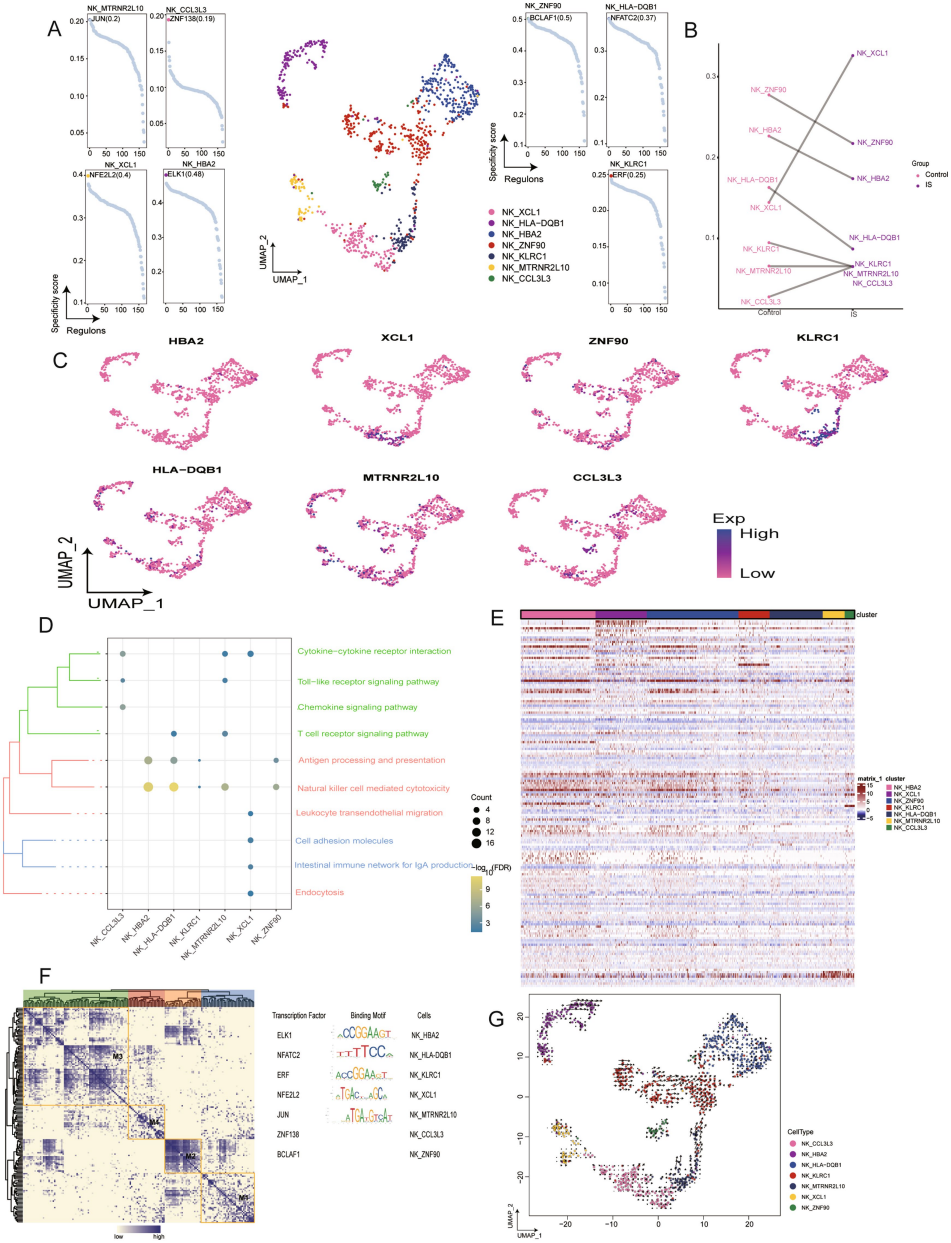
Identification of NK cell clusters in IS. **(A)** Single-cell plot of NK cell clusters and scatter plot subclusters. **(B)** Mapping of marker genes in NK cell subclusters. **(C)** Comparison of cell abundance between patients with IS and controls. **(D)** Biological pathway enrichment of NK cell subclusters, with darker colors indicating significant enrichment. Count represents the number of genes enriched in a certain pathway, and the larger number, the more genes were enriched in that pathway. -Log10 (FDR) represents the significance of enrichment differences, with darker colors indicated more significant enrichment differences. **(E)** Heatmap of differentially expressed genes in NK cell subclusters. **(F)** Heatmap of motif-based transcription factor (TF) gene regulatory networks for NK cell subclusters. **(G)** Mapping of NK cells and differentiation trajectories for monocytes, arrows represent the direction of differentiation, and the length of arrows represents the speed of differentiation. UMAP, Uniform Manifold Approximation and Projection.

NK cells were grouped into seven subclusters (Figure 6A), all present during IS. X-C motif chemokine ligand 1 (XCL1) and CCL3L3 were significantly enriched in IS, whereas the abundance of HLA-DQB1, HBA2, ZNF90, and KLRC1 was reduced (Figure 6B). Additionally, we determined the expression levels of these seven genes and found no significant differences compared to other cell subtypes (Figure 6C). Enrichment analysis demonstrated NK_HBA2 and NK_HLA-DQB1 were positively correlated with NK cell-mediated cytotoxicity, whereas NK_XCL1 was negatively associated with leukocyte transendothelial migration, cell adhesion molecules, the intestinal immune network for IgA production, and endocytosis. A relationship between microglia and NK cells was proposed based on analysis (Figure 6D; Supplemental Table S7). The expression heatmap depicted the expression status of specific genes across subpopulations (Figure 6E). The gene regulatory networks analysis identified ELK1, NFATC2, ERF, NFE2L2, and JUN as transcriptional regulators of the NK cell subclusters (Figure 6F). Furthermore, by analyzing the expression status of NK cells at different time points in IS with cell trajectory analysis, it was indicated that NK_KLRC1 would differentiate into NK_HBA2 and NK_MTRNR2L10, while the speed of developing into NK_HBA2 was relatively fast. (Figure 6G).

### 3.6 Cell communication in IS

**Figure 7:**
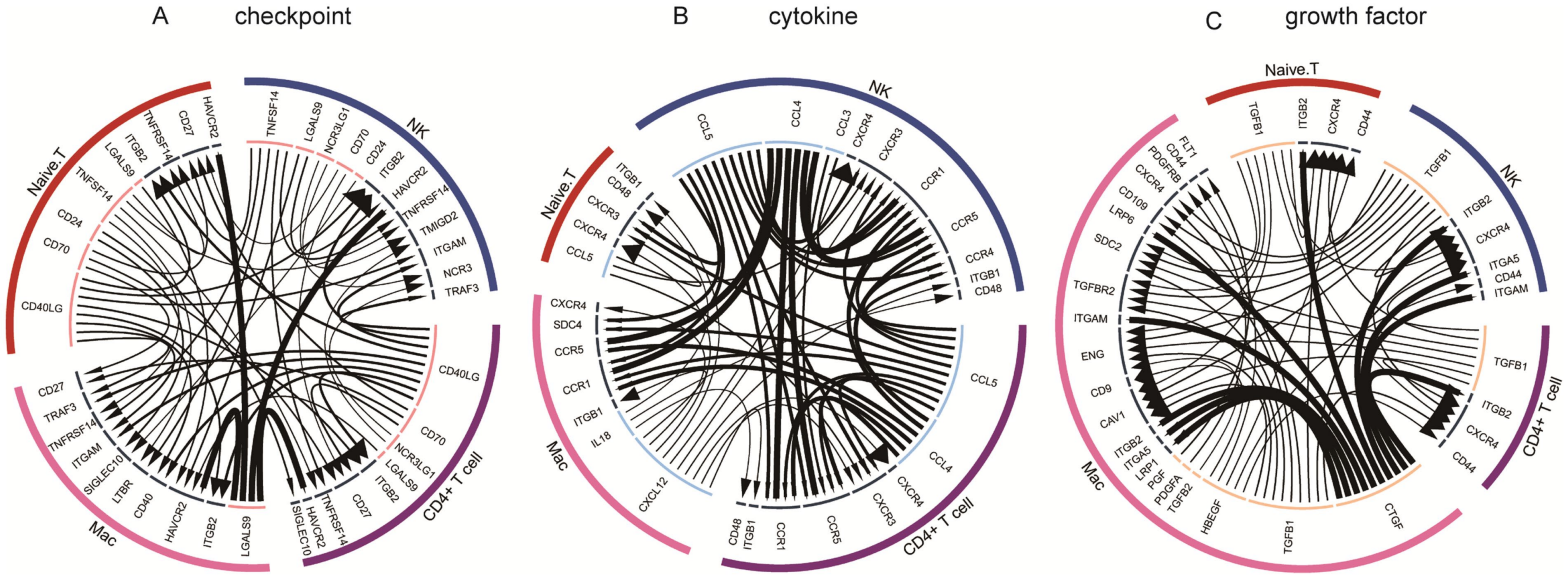
Cellular communication networks in IS subclusters. **(A)** Immune checkpoint modules involved in intracellular and intercellular communication. **(B)** Cytokine modules involved in intracellular and intercellular communication. **(C)** Growth factor modules involved in intracellular and intercellular communication.

Across the four subpopulations of CD4 + T cells, Mac, naïve T cells, and NK cells, overlapping gene expressions were observed. NBEAL1 was expressed in both CD4 + T cells and naïve T cells; MTRNR2L10 and ZNF90 were co-expressed in CD4 + T cells and NK cells; and RPS10 was common to both Mac and naïve T cells. Therefore, we evaluated the cellular interactions among the different modules of these four cell subpopulations. The immune checkpoint module (Figure 7A) revealed interactions between CCL5, CCR1, CXCL12, and CXCR4. The cytokine module (Figure 7B) showed the bi-directional influence of the CCL5-CCR1 and CXCL12-CXCR4 pathways on different cell types. However, the growth factor module (Figure 7C) highlighted the involvement of the CTGF-ITGAM pathway in regulating central nervous system function.

## 4 Discussion

This study utilized a single-cell analysis of public database to identify four cell types: CD4 + T cells, Mac, naïve T cells, and NK cells. We performed an enrichment analysis of these cell subpopulations, which revealed significant differences in the biological functions of these cell subclusters between the IS and control groups. Furthermore, we explored cell developmental trajectories and intercellular interactions, offering an in-depth understanding of the molecular mechanisms underlying IS and establishing a theoretical foundation for its pathology. Our findings indicated that the abundance of naïve T cells and NK cells increased during IS, whereas CD4 + T cells and Mac decreased. Notably, naïve T cells may disrupt tolerance to autoantigens in atherosclerosis, potentially influencing immune mechanisms and offering a preventive avenue for stroke (Khan et al., 2024). NK cells were observed to be more highly expressed in stable plaques than in unstable plaques (Wang et al., 2022).

Cerebral infarction can manifest in several types, with atherosclerosis being the most common cause of large-artery atherosclerotic infarctions. Unstable plaques associated with atherosclerosis are significant contributors to cerebral infarction, where NK cells activate autoimmune defense mechanisms during IS. CD4 + T cells, classified as T-helper cells, play diverse roles in lymphocyte-mediated immune responses. Studies have shown that brain regulatory T cells inhibit astrocyte proliferation and enhance neurological recovery during stroke (Yuan et al., 2023; Ito et al., 2019). In this study, we observed a reduction in CD4 + T cells during cerebral infarction, aligning with the results of previous studies. The phagocytic activity of macrophages contributes to cerebral infarction by causing brain damage and disrupting neurological function. Studies have shown that suppressing macrophage phagocytosis during the IS can improve neurobehavioral outcomes and reduce brain damage (Shi et al., 2021). The levels of macrophages vary in different stages of IS, in the early stages, macrophage has the same upward trend as well as both neutrophils and microglia, while they begin to decrease around 7 days. Interestingly, the number of Mac in this study decreased, which may be related to the inclusion criteria and sampling (Fernandez et al., 2019; Zhang et al., 2022). This also indicates that it is pivotal to treat acute IS. Collectively, these results highlight the critical role of these cell types in the pathogenesis of IS.

Our research further uncovered diverse biological functions of these cell subclusters in blood samples. CD4 + T cell subpopulations exhibited distinct enrichment patterns: CD4 + T cell_MTRNR2L1 was significantly enriched in oxidative phosphorylation, cell adhesion molecules, and antigen processing and presentation pathways, whereas CD4 + T cell_ZNF90 was predominantly associated with the chemokine signaling, Toll-like receptor, and MAPK signaling pathways. Previous studies have confirmed the involvement of oxidative phosphorylation in various neurodegenerative diseases, including cerebral infarction, Alzheimer’s, Huntington’s, and Parkinson’s diseases (Cabral-Costa and Kowaltowski, 2020). Mitochondrial dysfunction and increased oxidative stress can cause an insufficient energy supply, hypoxia, ischemia, and abnormal reperfusion responses, thereby contributing to disease pathogenesis (Ham and Raju, 2017). Specifically, oxidative phosphorylation exacerbates cerebral infarction (Anderson et al., 2013). Chemokine expression, distribution, and function are primarily associated with neuropathic pain. Chemokine signaling-mediated neuroinflammation has been linked to neuropathic pain and may also contribute to neurological deficits such as paresthesia during ischemic events (Zhang et al., 2017). Similarly, the MAPK signaling pathway accelerates inflammatory responses, oxidative stress, neuronal damage, and cognitive impairment during ischemic events (Tian et al., 2020; Xiang et al., 2020). These results suggested that with the increasing number of CD4 + T cell_MTRNR2L1 or CD4 + T cell_ZNF90, IS may get worse.

Interestingly, there were two differentiation trajectories in the CD4 + T cell_MTRNR2L1 subpopulation. Previous studies suggest that MTRNR2L1 protects against cerebral ischemia/reperfusion (I/R) injury by inhibiting ERK activation (Xu et al., 2006; Zhu et al., 2022). And the methylation of the MTRNR2L8 promoter is related to stroke (Shen et al., 2019). Conversely, there are currently no studies about IGKC with stroke, which deserve further exploration. Consequently, it is speculated that CD4 + T cell_MTRNR2L1 regulates oxidative phosphorylation and causes insufficient energy supply may be related to MTRNR2L8 methylation, thereby affecting the progression of acute IS attacks.

In Mac, the Mac_MT1E subpopulation was enriched in pathways regulating the actin cytoskeleton, chemokine signaling, lysosomes, and vascular smooth muscle contraction. In naïve T cells, naïve T_CD4 was significantly enriched in lysosomal pathways, the intestinal immune network for IgA production, and antigen processing and presentation pathways. Both Mac_MT1E and naïve T_CD4 were significantly enriched in the lysosomal signaling pathway as negative regulators. Lysosomes play a key role in cellular homeostasis, development, and aging by mediating autophagy, endocytosis, phagocytosis, and macrocytosis (Yang and Wang, 2021). Neuronal autophagy during stroke, achieved through autophagosome-lysosome degradation, protects against metabolic disorders, ischemia/reperfusion, energy deficiency, neurotoxins, trauma, and inflammation (Ajoolabady et al., 2021; Chen et al., 2020). Studies have shown that impaired autophagy during cerebral ischemia can disrupt lysosomal function, altering Mac cytokine secretion, damaging synaptic ultrastructure, and worsening neuronal dysfunction and neurodegeneration (Zhang Z. et al., 2021; Zhang X. et al., 2021). Therefore, protecting lysosomal function emerges as a potential strategy to prevent stroke.

Studies have also shown that in mouse models, the upregulation of MT-1 and MT-2 expression levels can reduce neuronal neurotoxicity, thereby playing a neuroprotective role in mice models with focal cerebral ischemia (Liu et al., 2020). Conversely, increased CD4 + and CD8 + T lymphocytes can worsen neurological dysfunction in elderly mice post-stroke (Fernandez et al., 2019; Levard et al., 2024). Nevertheless, when we predicted the developmental trajectories of Mac and Naive. T cells, we found that the differentiation trajectories of Mac_MT1E and Naive.T_CD4 were not obvious. We considered that this may be related to differences in gene expression in the samples.

In NK cells, our research found that endocytosis was one of the pathways significantly enriched in the NK_XCL1 subgroup. Endocytosis is a widely observed cellular process. Numerous studies have shown that microglial phagocytosis plays an important role in IS (Jia et al., 2021). During acute cerebral ischemia, microglia phagocytose and clear neurons, ischemic necrotic cell fragments, endothelial cells, and leukocytes. Nonetheless, excessive phagocytosis can trigger secondary inflammatory responses or result in the excessive loss of neurons, aggravating neurological deficits (Candelario-Jalil et al., 2022; Planas, 2024; Chen et al., 2022). Some studies have found that activating negative regulators of NK cells can reduce NK cell dysfunction and protect the brain’s immune defense, which is crucial for preventing post-stroke infections (Feng et al., 2021). Dynamic changes in NK cells during stroke manifest as a reduced number and activity in the peripheral blood and increased infiltration in brain tissue (Qi and Liu, 2023). NK cells damage neurons through immune defense mechanisms, causing barrier dysfunction through cytotoxicity and inflammatory activity, thereby exacerbating stroke outcomes (Qi and Liu, 2023; Liu et al., 2017). Our study observed that the number of NK cells in the atherosclerotic plaque tissue of the internal carotid artery in cerebral infarction increased, and endocytosis was significantly enriched in the NK_XCL1 subpopulation. We speculate that NK cells may also play an endocytic role in the immune defense process, accelerating neuronal cell death or dissolution and aggravating cerebral infarction. However, there have been no relevant reports thus far, and further investigation is required. Moreover, in the cell differentiation trajectory, the two cell differentiation trajectories of NK cells subclusters we discovered have not been reported so far, and NK_XCL1 is also present in them, it is novel perspective for us to explore in the future.

Additionally, our study identified significantly dysregulated genes in cell subsets, revealing that NBEAL1 and XCL1 were significantly upregulated, whereas ARPC5 was significantly downregulated. Previous studies have shown that NBEAL1 regulates low-density lipoprotein (LDL) uptake. Low expression of NBEAL1 disrupts LDL levels, promoting atherosclerosis and inducing cerebral infarction (Bindesbøll et al., 2020). This indicated that NBEAL1 has a protective effect on IS. In a mouse model experiment, the upregulated expression of ARPC5 was found to have a protective effect on neuronal cells during cerebral infarction, potentially through endocytosis mechanisms (Wan et al., 2021). The role of XCL1 in stroke remains unreported and requires further investigation. These genes may serve as potential biomarkers for the early diagnosis of cerebral infarction. We evaluated cell communication interactions and identified key immune mechanisms. In the immune checkpoint module, LGALS9-HAVCR2 enhanced macrophage-mediated immune checkpoint activation and T cell exhaustion during disease progression (Yan et al., 2024), whereas CD40LG-ITGB2 activated inflammation-related pathways associated with internal carotid artery atherosclerosis and vascular elasticity (Schnabel et al., 2008; Shami et al., 2021). In the cytokine module, CCL5-CCR1 mediated inflammatory signaling, regulating stromal cells to promote tissue regeneration and repair nerve cells damaged by cerebral infarction (Kauts et al., 2013; Julián-Villaverde et al., 2022). CXCL12-CXCR4 mediates protective NK cell activity in the pathological process of ischemic brain injury, contributing to stroke recovery (Wang S. et al., 2023). The growth factor module revealed CTGF-ITGAM transduction. CTGF is involved in cerebral infarction angiogenesis, and inhibition of this pathway can improve recovery (George et al., 2018). The results of the cell communication revealed the inflammatory response in cerebral infarction and the clearance and protection of immune cells important for cerebral infarction recovery.

From our analysis, we intuitively demonstrated the single-cell landscape of immune cells in IS. In the cell population, XCL1, one of the different expression genes (DEGs), was identified for the first time, which may serve as a novel potential candidate gene for the diagnosis and treatment of IS. Moreover, NBEAL1 and ARPC5 are both candidate genes for protective effects, and enrichment analysis has showed the biological functions of cell subpopulations. In terms of signaling pathways, they were mostly enriched in negative regulatory signaling pathways such as oxidative phosphorylation, lysosomes, and endocytosis, which are meaningful for targeted therapy of IS. Additionally, our RNA velocity analysis simulated the trajectory of cell differentiation and predicted their fate, which may provide a new target for IS diagnosis and treatment. Last but not least, we also found that there was currently a mismatch between TFs and binding motifs, which means there are still many molecular mechanisms worth exploring and uncovering, as binding motif analysis is worthy of further study. However, this study has some limitations. It exclusively analyzed carotid artery atherosclerotic plaques from male participants without exploring other tissue sources, such as brain tissue. Therefore, future research should incorporate diverse tissue samples for confirmation and validation. Additionally, as this study relied primarily on bioinformatics analysis, experimental validation through cell and animal studies is imperative.

## 5 Conclusion

This study identified the biological functions and characteristics of the four cell subtypes in IS using single-cell analysis. The abundance of naïve T and NK cells increased in stroke patients, whereas that of CD4 + T cells and Mac decreased. Enrichment analysis revealed that cell subtypes enriched in oxidative phosphorylation, lysosome, and endocytosis signaling pathways, which were significant differences in the differentiation trajectories of cell subpopulations. Through differential gene expression analysis, three significantly differentially expressed genes (NBEAL1, XCL1, and ARPC5) were identified, and the transcription regulatory factors affecting the different cell subgroups were preliminarily identified. This could become the starting point for future diagnosis and treatment of cerebral infarction.

## Data Availability

The original contributions presented in the study are included in the article/Supplementary material, further inquiries can be directed to the corresponding authors.

## Footnotes

^1^ https://www.ncbi.nlm.nih.gov/geo/

^2^ http://www.bioinforcloud.org.cn

## Supplementary material

Supplementary Figure S1 Differences in cellular abundance components between patients with IS and the control group.

## Glossary

ARPC5: actin related protein 2/3 complex subunit 5
BP: biological processes
CC: cellular component
DEG: different expression gene
FDR: false discovery rate
GEO: Gene Expression Omnibus
GLM: generalized linear model
GSEA: Gene set enrichment analysis
IS: ischemic stroke
LDL: low-density lipoprotein
KEGG: Kyoto Encyclopedia of Genes and Genomes
MF: molecular function
Mac: macrophages
NK: natural killer
NGS: next generation sequencing
NBEAL1: neurobeachin like 1
scRNA-seq: single-cell RNA sequencing
SCENIC: Single-cell regulatory network inference and clustering
TFs: transcription factors
UMAP: Uniform manifold approximation and projection
XCL1: X-C motif chemokine ligand 1
