# Supplementary figures and images for: Decoding immune cell dynamics in ischemic stroke: insights from single-cell RNA sequencing analysis
Source: Front Aging Neurosci. 2025 Apr 15;17:1549518. doi: 10.3389/fnagi.2025.1549518 (PMC12037566; doi:10.3389/fnagi.2025.1549518)

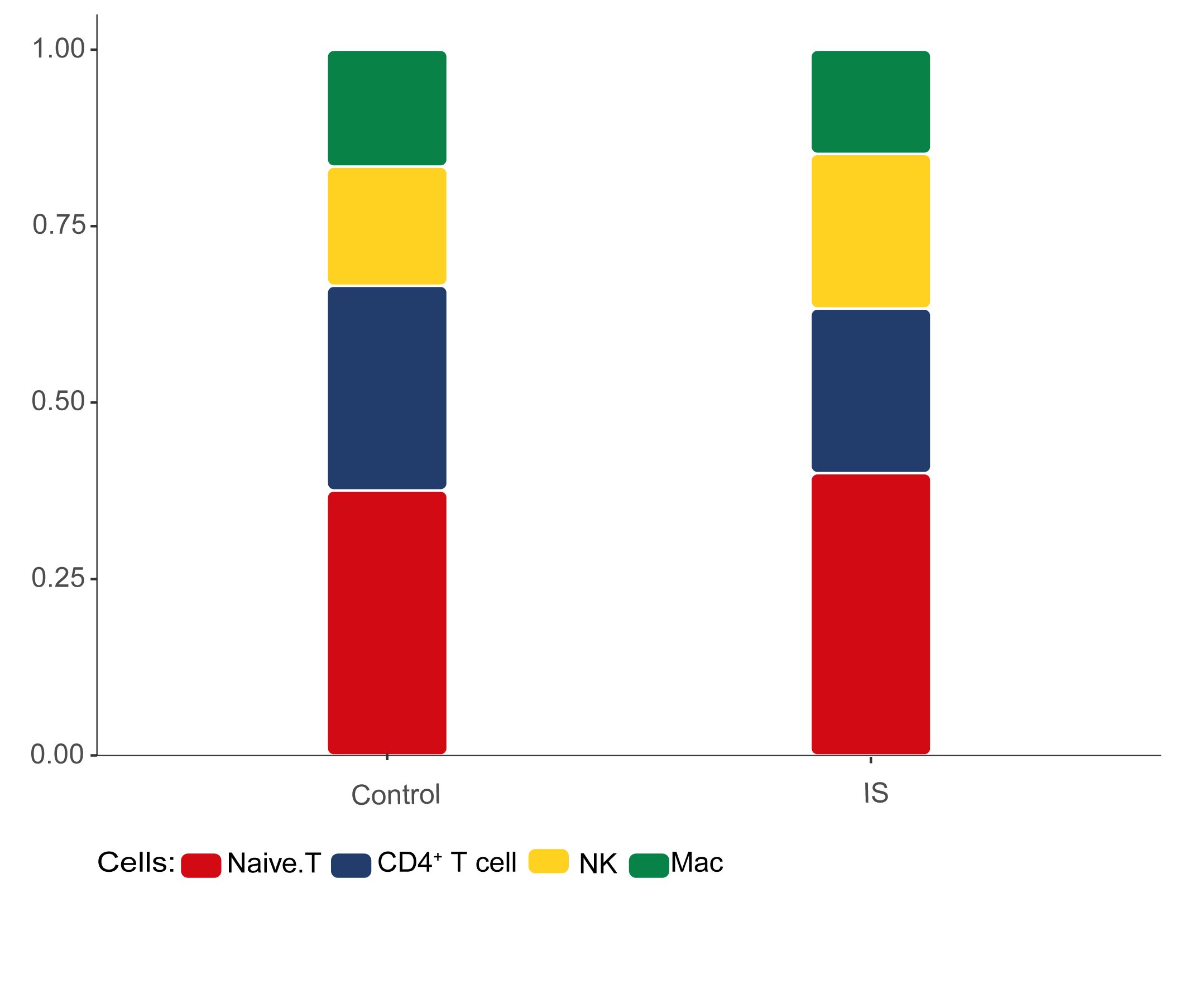

Supplement: Supplementary Figure S1 — Differences in cellular abundance components between patients with IS and the control group. [file Image_1.TIF]
